# Hereditary Hemochromatosis Predisposes Mice to *Yersinia pseudotuberculosis* Infection Even in the Absence of the Type III Secretion System

**DOI:** 10.3389/fcimb.2016.00069

**Published:** 2016-06-24

**Authors:** Halie K. Miller, Leah Schwiesow, Winnie Au-Yeung, Victoria Auerbuch

**Affiliations:** ^1^Department of Microbiology and Environmental Toxicology, University of California Santa CruzSanta Cruz, CA, USA; ^2^Department of Molecular, Cell, and Developmental Biology, University of California Santa CruzSanta Cruz, CA, USA

**Keywords:** *Yersinia pseudotuberculosis*, type III secretion system, IscR, hemochromatosis, HFE, hemojuvelin

## Abstract

The iron overload disorder hereditary hemochromatosis (HH) predisposes humans to serious disseminated infection with pathogenic *Yersinia* as well as several other pathogens. Recently, we showed that the iron-sulfur cluster coordinating transcription factor IscR is required for type III secretion in *Y. pseudotuberculosis* by direct control of the T3SS master regulator LcrF. In *E. coli* and *Yersinia*, IscR levels are predicted to be regulated by iron bioavailability, oxygen tension, and oxidative stress, such that iron depletion should lead to increased IscR levels. To investigate how host iron overload influences *Y. pseudotuberculosis* virulence and the requirement for the Ysc type III secretion system (T3SS), we utilized two distinct murine models of HH: hemojuvelin knockout mice that mimic severe, early-onset HH as well as mice with the *Hfe*^C282Y∕C282Y^ mutation carried by 10% of people of Northern European descent, associated with adult-onset HH. *Hjv*^−∕−^ and *Hfe*^C282Y∕C282Y^ transgenic mice displayed enhanced colonization of deep tissues by *Y. pseudotuberculosis* following oral inoculation, recapitulating enhanced susceptibility of humans with HH to disseminated infection with enteropathogenic *Yersinia*. Importantly, HH mice orally infected with *Y. pseudotuberculosis* lacking the T3SS-encoding virulence plasmid, pYV, displayed increased deep tissue colonization relative to wildtype mice. Consistent with previous reports using monocytes from HH vs. healthy donors, macrophages isolated from *Hfe*^C282Y∕C282Y^ mice were defective in *Yersinia* uptake compared to wildtype macrophages, indicating that the anti-phagocytic property of the *Yersinia* T3SS plays a less important role in HH animals. These data suggest that *Yersinia* may rely on distinct virulence factors to cause disease in healthy vs. HH hosts.

## Introduction

Iron is an essential element for almost all microorganisms, with the exception of some examples including *Lactobacillus plantarum* and *Borrelia burgdorferi* (Archibald, 1983; Posey and Gherardini, 2000). Most bacteria require anywhere from 10^−6^ to 10^−7^ M free iron to support growth. However, pathogenic bacteria often encounter iron-limiting conditions, particularly during growth within mammalian hosts due to the success of host iron sequestration systems (Weinberg, 1978; Cassat and Skaar, 2013). These systems include binding of iron to the storage protein ferritin, complexing iron with heme, and the tight association of serum iron to transferrin (Cassat and Skaar, 2013). Free iron in humans is further sequestered during infection via inflammation-induced hypoferremia, which includes host production of increased amounts of lactoferrin in an attempt to restrict bacterial growth (Jurado, 1997). To compensate, pathogens employ a number of iron acquisition mechanisms to acquire iron from the host, including siderophores, transferrin/lactoferrin receptors, heme acquisition systems, and other types of iron uptake systems (Cassat and Skaar, 2013).

The ability to sense the iron limiting environment of mammalian hosts not only allows for induction of bacterial iron acquisition systems, but serves as a signal for many pathogens to regulate virulence determinant expression (Skaar, 2010). An important virulence determinant for many Gram-negative pathogens that can be regulated by iron is the type III secretion system (T3SS; Murphy and Payne, 2007; Ellermeier and Slauch, 2008; Gode-Potratz et al., 2010; Chakraborty et al., 2011; Kurushima et al., 2012). This system utilizes a needle-like apparatus to deliver a series of effector proteins directly into host cells leading to modulation of normal host cell processes (Cornelis, 2006). *Shigella dysenteriae, Salmonella enterica, Vibrio parahaemolyticus, Bordetella bronchiseptica*, and *Edwardsiella tarda* are a few of the Gram-negative pathogens that regulate their T3SS in response to iron bioavailability within the host (Murphy and Payne, 2007; Ellermeier and Slauch, 2008; Gode-Potratz et al., 2010; Chakraborty et al., 2011; Kurushima et al., 2012). The *Yersinia* Ysc T3SS, which is encoded on the 70 kb virulence plasmid termed pYV, is modulated in response to temperature, calcium concentration, and host cell contact. Iron has never been demonstrated to modulate expression or function of the *Yersinia* Ysc T3SS and studies are typically performed under iron replete conditions (Cornelis et al., 1998). Recently, our group identified the iron-sulfur cluster coordinating transcription factor IscR as a novel component of the *Yersinia* T3SS regulatory cascade; deletion of IscR leads to a dramatic decrease in secretion of T3SS effector proteins (Miller et al., 2014). In that study, we demonstrated that IscR is essential for T3SS expression through direct regulation of *lcrF*, which encodes an AraC-type DNA binding protein responsible for expression of the majority of T3SS genes (Cornelis et al., 1998). It remains unclear exactly which environmental stimuli influence IscR target gene expression; however, the closely related *E. coli* IscR has been shown to respond to oxidative stress and oxygen limitation as well as iron starvation (Giel et al., 2006; Yeo et al., 2006; Wu and Outten, 2009).

The fact that IscR regulates the *Y. pseudotuberculosis* Ysc T3SS suggests that iron may play an important role in modulating expression of *Yersinia* virulence factors. Indeed, enteropathogenic *Yersinia* transit from the intestinal lumen, where they may be able to successfully compete for dietary iron, to severely iron restricted distal tissues. It is in these deeper tissue sites where the *Yersinia* Ysc T3SS has been shown to translocate effector proteins called Yops into cells such as macrophages and neutrophils (Marketon et al., 2005; Koberle et al., 2009; Durand et al., 2010). These Yops act to inhibit phagocytosis and to dampen inflammatory properties of innate immune cells (McCance and Widdowson, 1938; Martin et al., 1987; Miret et al., 2003; Heesemann et al., 2006; Matsumoto and Young, 2009). How host iron availability impacts T3SS utilization and virulence in *Yersinia* is unclear.

Iron overload disorders such as hereditary hemochromatosis (HH) predispose individuals to *Yersinia* infection (Jacquenod et al., 1984; Mennecier et al., 2001a; Harris and Paraskevakis, 2012; Quenee et al., 2012). Hereditary hemochromatosis (HH) is a genetic iron overload disorder and is one of the most common genetic disorders in Caucasians (Bahram et al., 1999). Individuals with HH absorb excess dietary iron, which then accumulates in tissues such as the liver. If left untreated, this iron accumulation can lead to organ failure as a result of iron-induced oxidative stress (MacKenzie et al., 2008). Mutations within several different genes, including the high iron Fe (*Hfe*) and the hemojuvelin (*Hjv*) genes, have been linked to HH. Both *Hfe* and *Hjv* act to control expression of the iron-regulating hormone hepcidin, which regulates iron uptake in the gut and the recycling of senescent red blood cells by macrophages (Feder et al., 1996; Bahram et al., 1999; Huang et al., 2005). The *Hfe* C282Y mutation is associated with adult-onset HH, while *Hjv* mutations are rare and associated with more severe, early onset iron overload (Brandhagen et al., 2002; Lanzara et al., 2004). The *Hfe*^C282Y∕C282Y^ and *Hjv*^−∕−^ mouse models have been developed for the study of HH and mimic a number of the symptoms seen in the human disease, including excess liver iron (Levy et al., 1999; Huang et al., 2005).

Quenee et al. previously showed that an *Hjv* mutation predisposes mice to infection with a *Y. pestis* vaccine strain lacking the *pgm* locus, which encodes the yersiniabactin siderophore iron uptake system. However, the authors observed no difference in the ability of fully virulent *Y. pestis* to cause disease in wildtype and *Hjv*^−∕−^ mice (Quenee et al., 2012). While humans with HH are at higher risk for contracting disseminated enteropathogenic *Yersinia* infection (Piroth et al., 1997; Bergmann et al., 2001; Hopfner et al., 2001; Mennecier et al., 2001b), whether *Hfe*^C282Y∕C282Y^ and *Hjv*^−∕−^ mice are more susceptible to *Y. pseudotuberculosis* is unknown. Additionally, based on the knowledge that IscR directly regulates T3SS expression, it is unclear to what extent the Ysc T3SS contributes to *Y. pseudotuberculosis* pathogenesis in an HH host. This work provides the first evidence for enhanced susceptibility of *Hfe*^C282Y∕C282Y^ and *Hjv*^−∕−^ mice to *Y. pseudotuberculosis*. Furthermore, we demonstrate that there is a decreased requirement for the *Yersinia* Ysc T3SS for disease causation in hosts with hereditary hemochromatosis.

## Materials and methods

All animal use procedures were in strict accordance with the NIH Guide for the Care and Use of Laboratory Animals and were approved by the UCSC Institutional Animal Care and Use Committee.

### Bacterial strains and growth conditions

*Y. pseudotuberculosis* strains used in this study are included in Table 1. Unless specified, *Y. pseudotuberculosis* strains were grown overnight in LB at 26°C with shaking at 250 rpm for animal infections. For macrophage infections, bacteria were grown overnight in 2 × YT at 26°C at 250 rpm, back-diluted to OD_600_ of 0.2 into low calcium medium (2 × YT with 20 mM sodium oxalate and 20 mM MgCl_2_), grown for 1.5 h at 26°C at 250 rpm, and then for 1.5 h at 37°C at 250 rpm to induce the T3SS (Auerbuch et al., 2009).

**Table 1:** ***Y. pseudotuberculosis* strains used in this study**.

**Strain**	**Background**	**Mutation(s)**	**References**
WT	IP2666	pYV+, Naturally lacks full-length YopT	Bliska et al., 1991
Δyop6	IP2666	Δ*yopHEMOJ*	Auerbuch et al., 2009
pYV-	IP2666	Δ*yscBL* pYV cured	Auerbuch et al., 2009
Δyop6/Δ*yopB*	IP2666	Δ*yopHEMOJ* Δ*yopB*	Auerbuch et al., 2009

### Mouse infections

*Hfe*^C282Y∕C282Y^ mice were rederived in the UC Santa Cruz vivarium and *Hjv*^−∕−^ breeding pairs were obtained from Dr. Nancy Andrews (Duke University). Colonies were maintained through a combination of mating pairs including homozygous knockout/homozygous knockout, heterozygous mutant/heterozygous mutant as well as homozygous knockout/heterozygous mutant. Age matched wildtype mice were obtained through wildtype/wildtype as well as the above mentioned heterozygous mutant/heterozygous mutant matings. Genotypes were determined from tail biopsies processed using the DNeasy Blood & Tissue Kit (Qiagen) per the manufacturer's protocol. Wildtype or knockout female and male, 11 to 12-week-old *Hfe*^C282Y∕C282Y^ mice and 5 to 12-week-old *Hjv*^−∕−^ mice in the 129S6/SvEvTac background from our breeding facilities were used for oral infections as previously described (Auerbuch and Isberg, 2007). Mice were orogastrically inoculated with 2 × 10^8^ colony forming units (CFU) for *Hfe*^C282Y∕C282Y^ infections or 2 × 10^7^ CFU for *Hjv*^−∕−^ infections in a 200 μl volume of PBS using a feeding needle. Mice were given food containing a standard amount of iron (200 ppm) and water *ad libitum* and were euthanized at either 3 or 5 days post-inoculation. Peyer's patches, mesenteric lymph nodes (MLN), spleens, and livers were isolated and homogenized for 30 s in PBS followed by serial dilution and plating on LB supplemented with 1 μg mL^−1^ irgasan for CFU determination.

### Tissue iron content

Total hepatic iron content was measured in tissue homogenates (tissue homogenized in PBS containing 0.2% NP-40, 1:4 w/v tissue:buffer) processed for analyses by aliquoting into acid-cleaned polyethylene tubes, evaporating to dryness, and digesting in 16N quartz-distilled HNO_3_ (Optima, Fisher Scientific) at 80°C in a heat block. Following complete digestion, samples were diluted with Milli-Q water (18 Mohm/cm^2^) for analyses; rhodium was added as an internal standard. Iron levels were measured by high resolution inductively coupled plasma–mass spectrometry (Thermo Scientific Element XR ICP-MS), measuring masses ^54^Fe, ^56^Fe, ^57^Fe, and ^103^Rh. The analytical detection limit and measurement precision was 5.68 ng/mL 1.04% RSD, respectively.

### Peritoneal macrophages

Peritoneal macrophages were isolated from 129S6/SvEvTac wildtype, *Hfe*^C282Y∕C282Y^, and *Hjv*^−∕−^ mice as described in Layoun et al. (2015). Briefly, mice were injected with 1 mL of 3.8% Brewer's thioglycollate media (BD Biosciences). Four days post-injection, mice were euthanized and peritoneal macrophages isolated. Macrophages were seeded at 5 × 10^5^ cells/well into a 24 well plate and allowed to adhere to the plate for 2 h or overnight. Macrophages were then treated with 100 ng ml^−1^ of lipopolysaccharide (LPS) from *Salmonella minnesota* (UltraPure), or exposed to either a *Y. pseudotuberculosis* mutant lacking Yop effectors but otherwise expressing a functional T3SS (T3SS+, Δyop6; Auerbuch et al., 2009) or with T3SS translocon deficient *Y. pseudotuberculosis* (T3SS-, Δyop6/Δ*yopB*) at an MOI of 10. Macrophages were treated for 3 h, at which time supernatants were collected and analyzed for cytokine levels as described below.

### Inside/outside staining

Peritoneal macrophages were isolated as described above. After being allowed to adhere to coverslips for 2 h, macrophages were infected with pYV- *Y. pseudotuberculosis* at an MOI of 10. After 0.5 h, infected cell monolayers were fixed for 10 min with 4% paraformaldehyde. Infected macrophages were treated with rabbit anti-*Yersinia* antibody (1:500), generously provided by Dr. Ralph Isberg (Tufts University), for 40 min at 37°C followed by treatment with goat anti-rabbit AlexaFlour 594 antibody (1:100, Life Technologies) for 40 min at 37°C. Macrophages were washed and then treated with ice cold methanol for 10 s to permeabilize. After permeabilizaton, macrophages were treated with rabbit anti-*Yersinia* antibody (1:500) for 40 min at 37°C followed by treatment with goat anti-rabbit FITC antibody (1:100, Santa Cruz Biotech) and Hoescht stain (1:10,000) for 40 min at 37°C. Coverslips were then mounted onto slides with Prolong Gold Antifade Reagent (Life Technologies). Slides were visualized on a Leica SP5 confocal microscope using Leica Application Suite Advanced Fluorescence software. Nine frames were obtained for each condition, and for each frame, the number of red bacteria and the number of green bacteria were counted using FIJI ImageJ software.

### ELISA cytokine measurement

Cytokine measurements were performed as previously described (Auerbuch and Isberg, 2007). Briefly, liver homogenates from *Hfe*^C282Y∕C282Y^ and wildtype mice, either uninfected or orogastrically inoculated with 2 × 10^8^ CFU of wildtype *Y. pseudotuberculosis*, were thawed on ice and centrifuged at 13,000 rpm for 1 min. The mouse inflammatory cytometric bead array kit (BD Biosciences) was used to detect IL-12p70, TNF-α, IFN-γ, MCP-1, IL-10, and IL-6 according to the manufacturer's protocol with the following exceptions. The amount of antibody-conjugated beads was decreased to 4 μl each with 20 μl of sample/standard and 20 μl of detection reagent per reaction. Data were acquired and analyzed using a BD FACS LSRII flow cytometer and BD analysis software, respectively. Cytokine levels detected in the livers of uninfected mice (3 *Hfe*^C282Y∕C282Y^ and 3 wildtype) were averaged and the standard deviations calculated for each cytokine tested (Excel). Standard deviations were added to the averages to determine the baseline cytokine level for uninfected livers. Individual cytokine concentrations in pg per ml^−1^ from infected samples were plotted against CFU per gram of liver tissue determined at the time of organ harvest.

### Statistical methods

All statistical methods in this study were analyzed using Kaleidagraph v4.1.1 for Windows (Synergy Software). Oral gavage infection studies were analyzed using the unpaired Wilcoxon–Mann–Whitney rank sum test. Measurement of hepatic iron load from tissues and measurement of cytokine levels from liver homogenates and peritoneal macrophages were analyzed using a Student's *t*-test. The correlation between bacterial burden and cytokine production was analyzed using Pearson's coefficient. Lastly, Student *t*-test was used for analysis of the uptake assay. Statistical significance for all analyses in this study was defined as *p* ≤ 0.05.

## Results

### Host mutations associated with iron overload, *Hfe*^C282Y∕C282Y^and *Hjv*^−∕−^, lead to enhanced systemic colonization of *Y. pseudotuberculosis*

**Figure 1:**
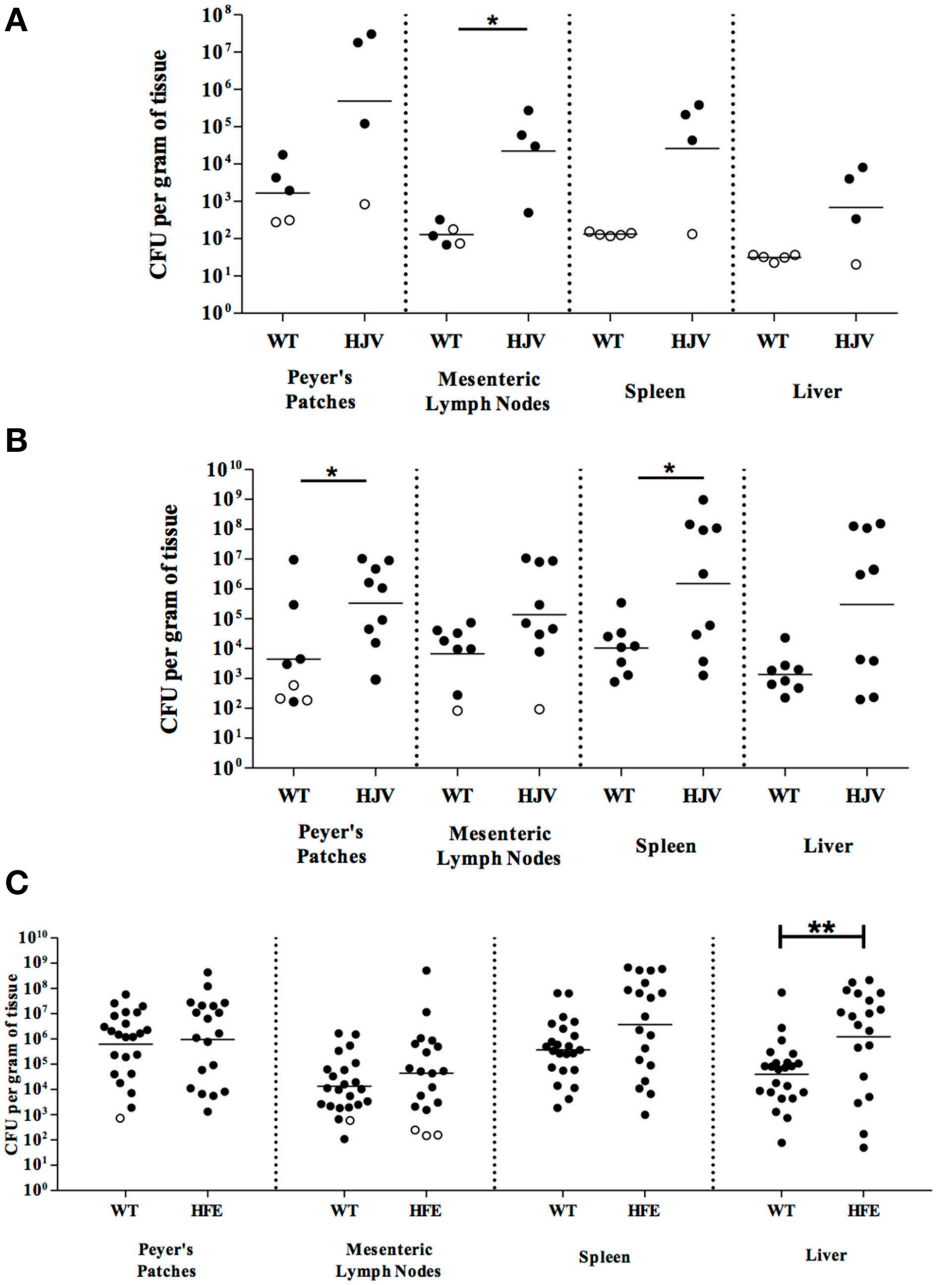
**Iron overload leads to enhanced colonization of mice orally infected with ***Y. pseudotuberculosis*****. Wildtype (WT) and *Hjv*^−∕−^ (HJV) mice were infected with 2 × 10^7^ WT *Y. pseudotuberculosis* via orogastric gavage. At **(A)** 3 days and **(B)** 5 days post-inoculation, the Peyer's patches, mesenteric lymph nodes, spleens and livers were collected, homogenized, and CFU determined. **(C)** WT and *Hfe*^C282Y∕C282Y^ (HFE) mice were infected with 2 × 10^8^ WT *Y. pseudotuberculosis* via orogastric gavage and tissues harvested 5 days post-inoculation. Each symbol represents data from one organ. Open symbols are set at the limit of detection for each individual organ based on weight and represent CFU that were below this limit. Dashes represent the geometric mean. Shown are data from **(A)** two, **(B)** four, and **(C)** seven independent experiments. ^*^p < 0.05, ^**^p < 0.01 as determined by an unpaired Wilcoxon–Mann–Whitney rank sum test. Dashes represent the geometric mean.

In order to better understand the biological significance of the influence of iron on *Y. pseudotuberculosis* pathogenesis, we studied susceptibility of murine models of hereditary hemochromatosis (HH) to *Y. pseudotuberculosis* infection. We began by evaluating the susceptibility of two distinct mouse models of HH, *Hfe*^C282Y∕C282Y^ and *Hjv*^−∕−^, to *Y. pseudotuberculosis* oral infection. While all wildtype mice survived for 5 days (when the experiment was terminated) following an oral inoculation dose of 2 × 10^8^ *Y. pseudotuberculosis*, 100% of *Hjv*^−∕−^ mice receiving the same dose had to be euthanized prior to day 5 because of symptoms indicative of more severe disease including hunched appearance and ruffled fur (unpublished observations), in accordance with institutional guidelines. This indicates that *Hjv*^−∕−^ mice may be more susceptible to oral infection with *Y. pseudotuberculosis* than normal mice. Using a lower oral inoculation dose of 2 × 10^7^ *Y. pseudotuberculosis* allowed both wildtype and *Hjv*^−∕−^ mice to survive 5 days of infection. We observed some differences in *Y. pseudotuberculosis* colonization of wildtype and *Hjv*^−∕−^ Peyer's patches and MLN 3 and 5 days post-inoculation (Figures 1A,B). However, more strikingly, while bacterial loads were below the limit of detection from the spleens and livers of all wildtype mice 3 days post-inoculation, the majority of *Hjv*^−∕−^ mouse spleens and livers contained greater numbers of bacteria relative to wild type (Figure 1A). Furthermore, by 5 days post-inoculation, *Hjv*^−∕−^ mice had 215- and 380-fold more CFU per gram tissue, on average, in the spleen and liver compared to wildtype mice (Figure 1B). Collectively, these data suggest that *Y. pseudotuberculosis* colonize deep tissues earlier in *Hjv*^−∕−^ mice compared to normal mice following the natural oral route of infection.

**Figure 2:**
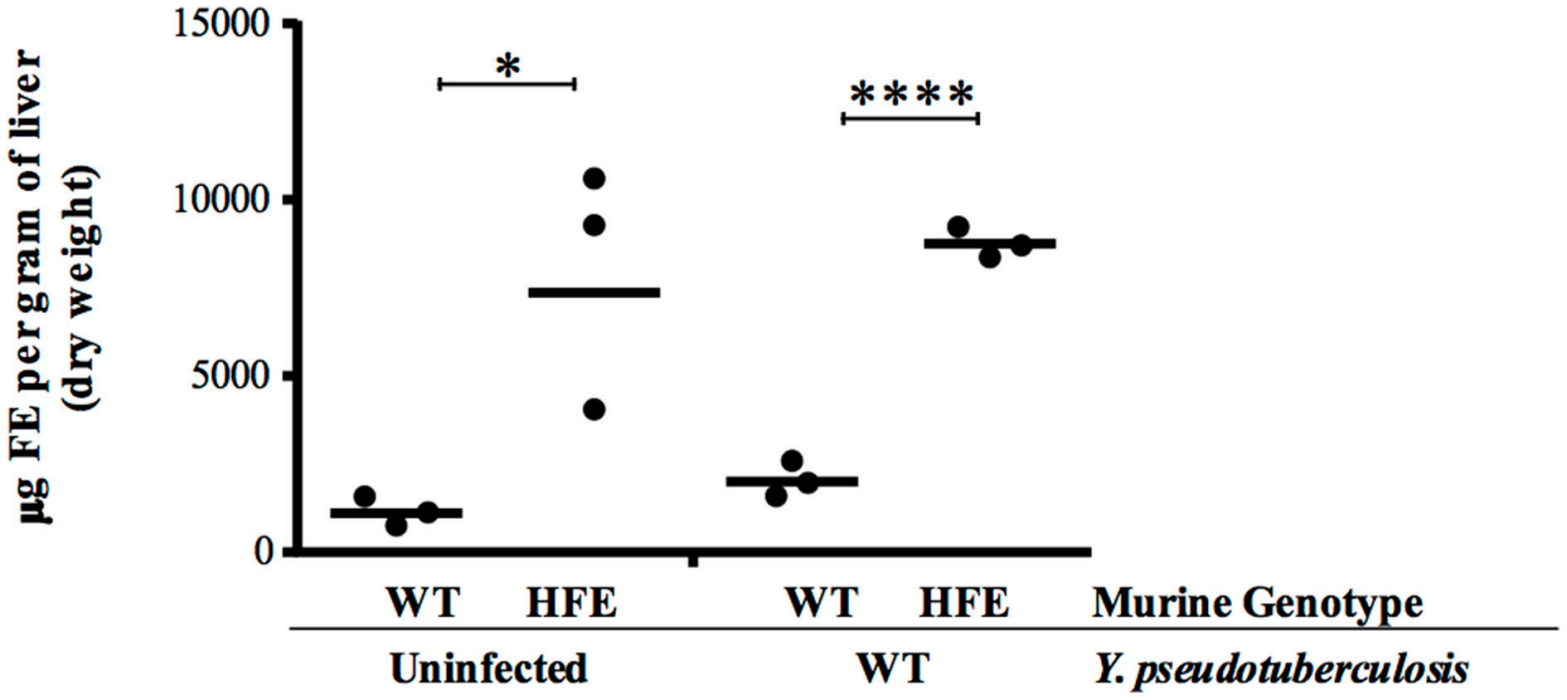
*****Hfe***^**C282Y∕C282Y**^ mice harbor increased hepatic iron**. Hepatic iron load was measured from tissues isolated from WT and *Hfe*^C282Y∕C282Y^ (HFE) mice that were left uninfected or were infected 5 days earlier with 2 × 10^8^ WT *Y. pseudotuberculosis* via orogastric gavage. Each symbol represents data from one organ. ^*^p < 0.05 and ^****^p < 0.0001 as determined by a Student *t*-test.

Analysis of the *Hfe*^C282Y∕C282Y^ mice, which display less severe iron overload relative to the *Hjv*^−∕−^ background (Huang et al., 2005), showed no significant differences in bacterial colonization 3 days post-inoculation compared to wildtype mice (data not shown) and comparable levels of colonization in the Peyer's patches, MLN, and spleens relative to wildtype mice days post-inoculation with 2 × 10^8^ *Y. pseudotuberculosis* (Figure 1C). However, colonization of the liver 5 days post-inoculation was increased in *Hfe*^C282Y∕C282Y^ mice by 30-fold (*p* < 0.01). This increased liver colonization of *Hfe*^C282Y∕C282Y^ mice correlated with four-fold more iron in *Hfe*^C282Y∕C282Y^ livers relative to wildtype livers during *Yersinia* infection (Figure 2).

Collectively, these data suggest that both the *Hfe*^C282Y∕C282Y^ and *Hjv*^−∕−^ mice are effective models for the study of *Y. pseudotuberculosis* infection in iron overloaded hosts. Based on the knowledge that the *Hfe*^C282Y∕C282Y^ mutation is far more common in humans than mutations in the *Hjv* gene, and that the *Hjv*^−∕−^ animals were significantly more challenging to breed, we focused the remainder of our studies on the *Hfe*^C282Y∕C282Y^ mouse model (Brandhagen et al., 2002; Lanzara et al., 2004).

### *Y. pseudotuberculosis* lacking the T3SS encoding virulence plasmid pYV display enhanced virulence in *Hfe*^C282Y∕C282Y^mice

**Figure 3:**
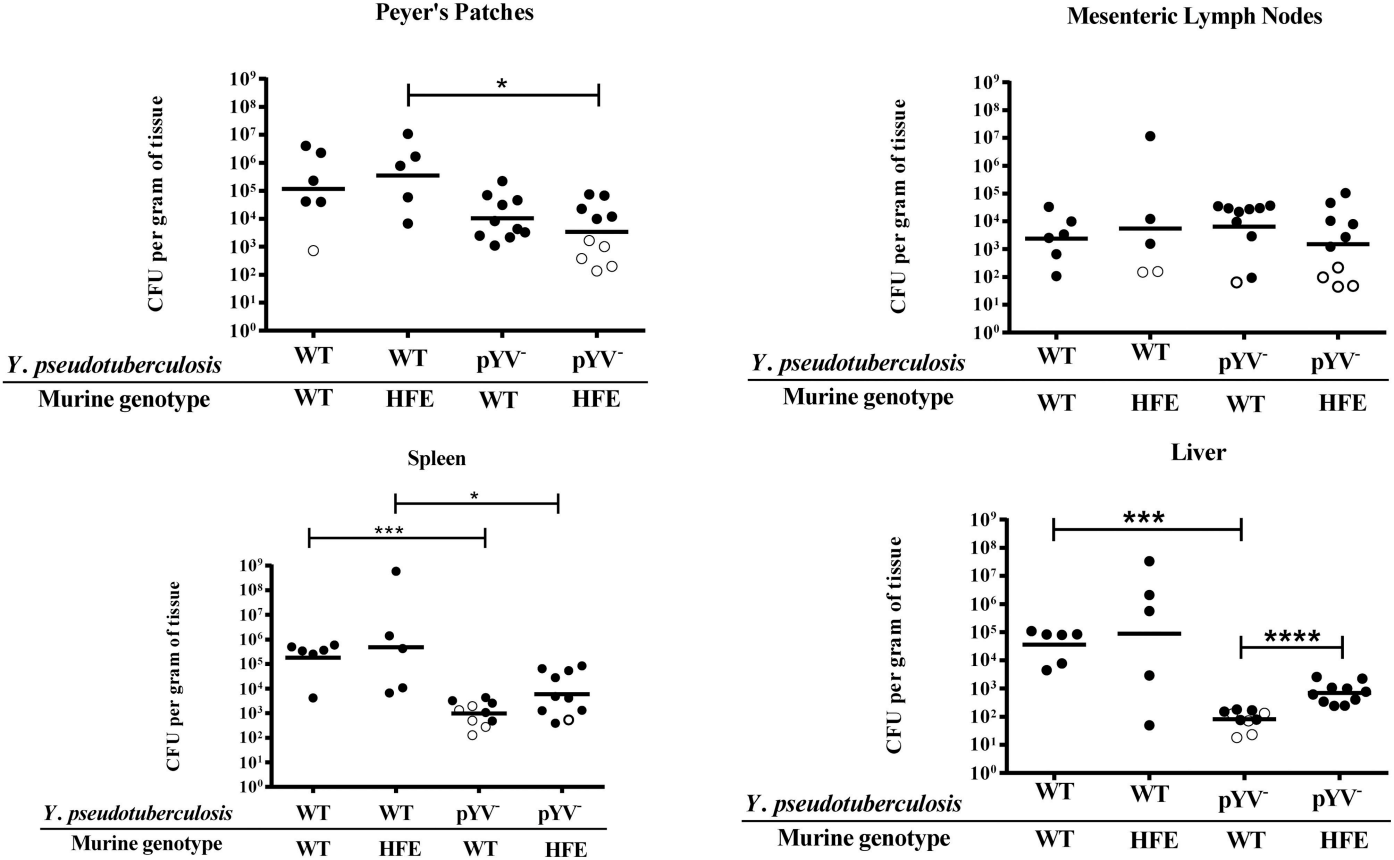
*****Y. pseudotuberculosis*** lacking the T3SS encoding virulence plasmid, pYV, are able to better colonize the livers of ***Hfe***^**C282Y∕C282Y**^ mice relative to wildtype mice**. WT and *Hfe*^C282Y∕C282Y^ (HFE) mice were infected with 2 × 10^8^ CFU of either WT *Y. pseudotuberculosis* or the pYV^−^ strain via orogastric gavage. At 5 days post-inoculation, the Peyer's patches, mesenteric lymph nodes, spleens and livers were collected, homogenized, and CFU determined. Each symbol represents data from one organ. Open symbols are set at the limit of detection for each individual organ based on weight and represent CFU that were below this limit. Dashes represent the geometric mean. Shown are data from three independent experiments. The wildtype data presented here is also included in Figure 1C. ^*^p < 0.05, ^***^p < 0.001, and ^****^p < 0.0001 as determined by an unpaired Wilcoxon–Mann–Whitney rank sum test.

Excess iron should lead to decreased IscR levels, and therefore decreased expression of the T3SS (Miller et al., 2014). Yet while the Ysc T3SS is required for *Yersinia* virulence in normal mice (Cornelis, 2002), we observed increased susceptibility of iron overloaded mice to *Y. pseudotuberculosis* (Figure 1). Thus, we evaluated the susceptibility of *Hfe*^C282Y∕C282Y^ mice to *Y. pseudotuberculosis* lacking the T3SS-encoding virulence plasmid, pYV. The pYV^−^ strain was better able to colonize livers of *Hfe*^C282Y∕C282Y^ mice compared to wildtype mice; pYV^−^ colonization of *Hfe*^C282Y∕C282Y^ livers was increased eight-fold (*p* < 0.0001) compared to pYV^−^ infection of wildtype tissues (Figure 3). In fact, while the pYV^−^ strain was able to colonize the liver to levels above the limit of detection in only 50% of the wildtype animals by day 5, this T3SS-deficient strain was able to colonize deep tissues to levels above the limit of detection in 95% of *Hfe*^C282Y∕C282Y^ mice. These findings suggest that there may be a decreased requirement for the *Y. pseudotuberculosis* Ysc T3SS in iron overloaded animals.

### Peritoneal macrophages from HH mice are not attenuated in their cytokine and chemokine response to *Yersinia*

**Figure 4:**
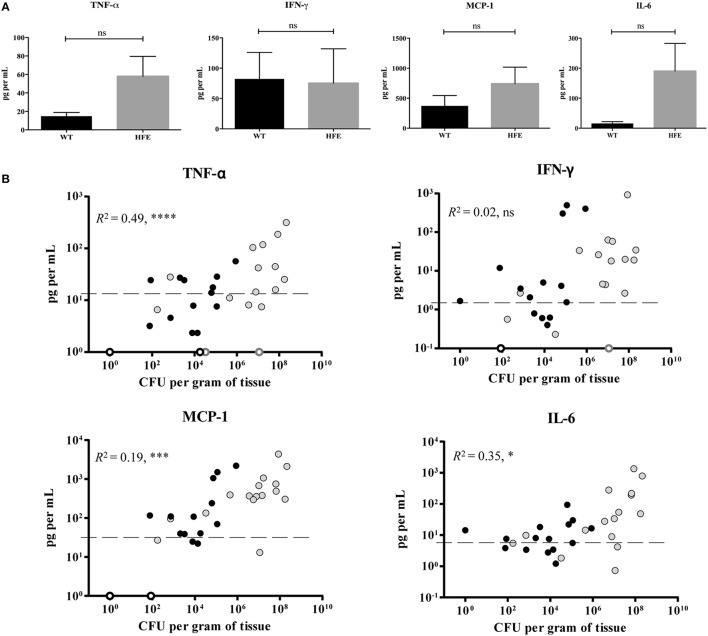
*****Hfe***^**C282Y∕C282Y**^ mice display robust hepatic cytokine production during ***Y. pseudotuberculosis*** infection**. WT (black) and *Hfe*^C282Y∕C282Y^ (HFE; gray) mice were infected with 2 × 10^8^ WT *Y. pseudotuberculosis* via orogastric gavage and tissues harvested 5 days post-inoculation. Flow cytometry-based ELISA was used to measure levels of the cytokines IFN-γ, IL-6, and TNF-α and the chemokine MCP-1 from liver homogenates. **(A)** IFN-γ, IL-6, TNF-α, and MCP-1 levels are displayed as mean pg per ml liver homogenate ± standard error of the mean; ns = no significance as determined by a Student *t*-test. **(B)** Individual cytokine concentrations in pg/ml are plotted against CFU per gram of liver tissue determined at the time of organ harvest. Open circles represent samples where CFU were below the limit of detection. Average uninfected cytokine concentrations are displayed as a dashed line and represent the average plus the standard deviation for uninfected liver samples. *R*^2^-values were determined based on Pearson's correlation coefficient using combined WT and HFE data; ns, no significance, ^*^p < 0.05, ^***^p < 0.001, and ^****^p < 0.0001.

Previous work by Wang et al., demonstrated that peritoneal macrophages isolated from *Hfe*^−∕−^ mice were defective in their cytokine response to *Salmonella* through Toll-like receptor 4, as a result of decreased cytokine mRNA translation (Wang et al., 2009). Several *Yersinia* T3SS effector proteins are known to inhibit production of several cytokines (Pha and Navarro, 2016). Therefore, we sought to examine whether cytokine production was decreased in HH mice during *Yersinia* infection, as diminished cytokine production may account for both the increased bacterial burden as well as the decreased requirement for the T3SS observed for these mice. We measured the levels of six different cytokines, TNF-α, IFN-γ, IL-6, IL-10, IL-12p70, and MCP-1, in *Yersinia*-infected mouse tissues. We found levels of IL-10 and IL-12p70 for both wildtype and *Hfe*^C282Y∕C282Y^ mice to be comparable to those of our uninfected controls (data not shown). These findings are not surprising as previous reports demonstrate these cytokines to be present at low levels until very late stages of infection (Auerbuch and Isberg, 2007). Liver TNF-α, IFN-γ, MCP-1, and IL-6 levels were above background in both *Hfe*^C282Y∕C282Y^ and wildtype mice and TNF-α, MCP-1, and IL-6 levels correlated with bacterial burden (Figures 4A,B; Pearson correlation). When WT and *Hfe*^C282Y∕C282Y^ liver cytokine levels were averaged, no significant differences could be detected (Figure 4A). In fact, consistent with many HH livers having higher average CFU burdens than wildtype mice, cytokine levels for a number of the HH livers were actually higher than for wildtype livers (Figure 4B).

**Figure 5:**
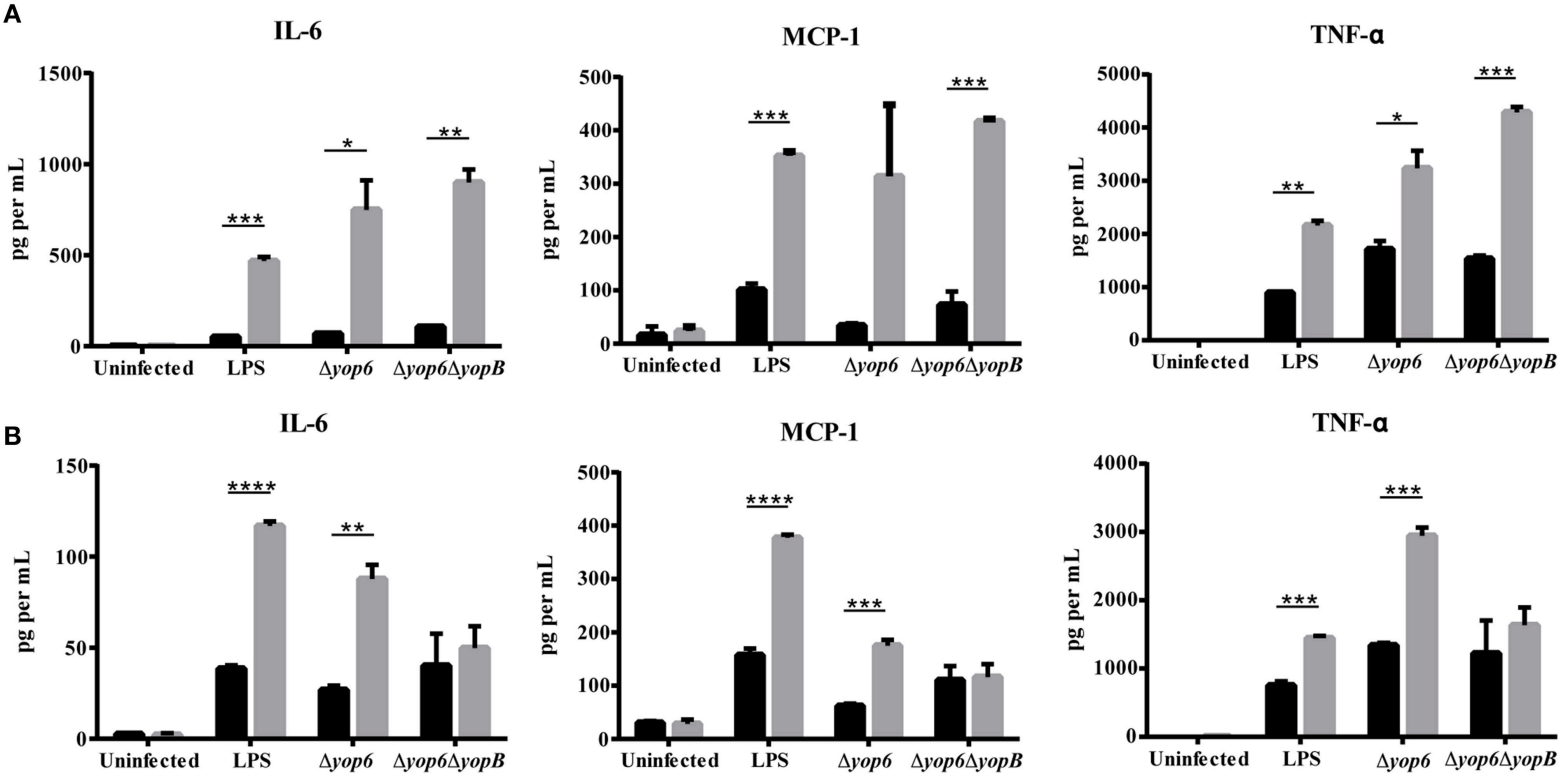
**Peritoneal macrophages isolated from hemochromatosis mice produce elevated levels of IL-6, MCP-1, and TNF-α in response to ***Y. pseudotuberculosis*** compared to macrophages from wildtype mice**. Elicited peritoneal macrophages from naïve WT (black bars), **(A)** *Hfe*^C282Y∕C282Y^ (gray bars), and **(B)** *Hjv*^−∕−^ (gray bars) mice were either left untreated, treated with 100 ng ml^−1^ of lipopolysaccharide (LPS) from *Salmonella minnesota* (UltraPure), or exposed to either Δyop6, a *Y. pseudotuberculosis* mutant lacking Yop effectors but expressing a functional T3SS translocon, or with Δyop6/Δ*yopB*, a T3SS translocon-deficient *Y. pseudotuberculosis* at an MOI of 10. Flow cytometry-based ELISA was used to measure levels of the cytokines IL-6 and TNF-α and the chemokine MCP-1 in the supernatant after 3 h. Data is displayed as the average cytokine concentration from the macrophages of two mice that had been pooled and analyzed in triplicate. ^*^p < 0.05, ^**^p < 0.01, ^***^p < 0.001, ^****^p < 0.0001 as determined by a Student *t*-test.

Because CFU burden differences between wildtype and HH mice complicated our cytokine analysis, we isolated thioglycollate-elicited peritoneal macrophages (which serve as a model for tissue macrophages) from wildtype, *Hfe*^C282Y∕C282Y^, and *Hjv*^−∕−^ mice, and infected them with *Y. pseudotuberculosis* either expressing a functional T3SS translocon but lacking the known T3SS effector proteins (Δyop6) or lacking a functional T3SS translocon (Δyop6/Δ*yopB*). These strains were used because several T3SS effector proteins have been shown to modulate cytokine production upon translocation inside host cells (Bliska et al., 2013). Surprisingly, the amount of IL-6, MCP-1, and TNFα secreted by *Hfe*^C282Y∕C282Y^ or *Hjv*^−∕−^ macrophages after 3 h was significantly higher than that secreted by wildtype macrophages in response to the Δyop6 or Δyop6/Δ*yopB Y. pseudotuberculosis* strains or to LPS (Figures 5A,B). Similar results were seen for the cytokine response to the WT and pYV- *Yersinia* strains (data not shown). Furthermore, incubating peritoneal macrophages for only 2 h following isolation and prior to inoculation, rather than overnight, did not alter these findings (data not shown). These data are in contrast to results from *Hfe*^−∕−^ C57Bl/6 mouse peritoneal macrophages treated with LPS or *Salmonella* (Wang et al., 2009), for reasons that remain unclear. However, these data suggest that an attenuated HH cytokine response is not responsible for the increased bacterial burden or decreased requirement for the T3SS during *Yersinia* infection of HH mice.

### Peritoneal macrophages from HH mice are defective in their ability to take up *Yersinia*

**Figure 6:**
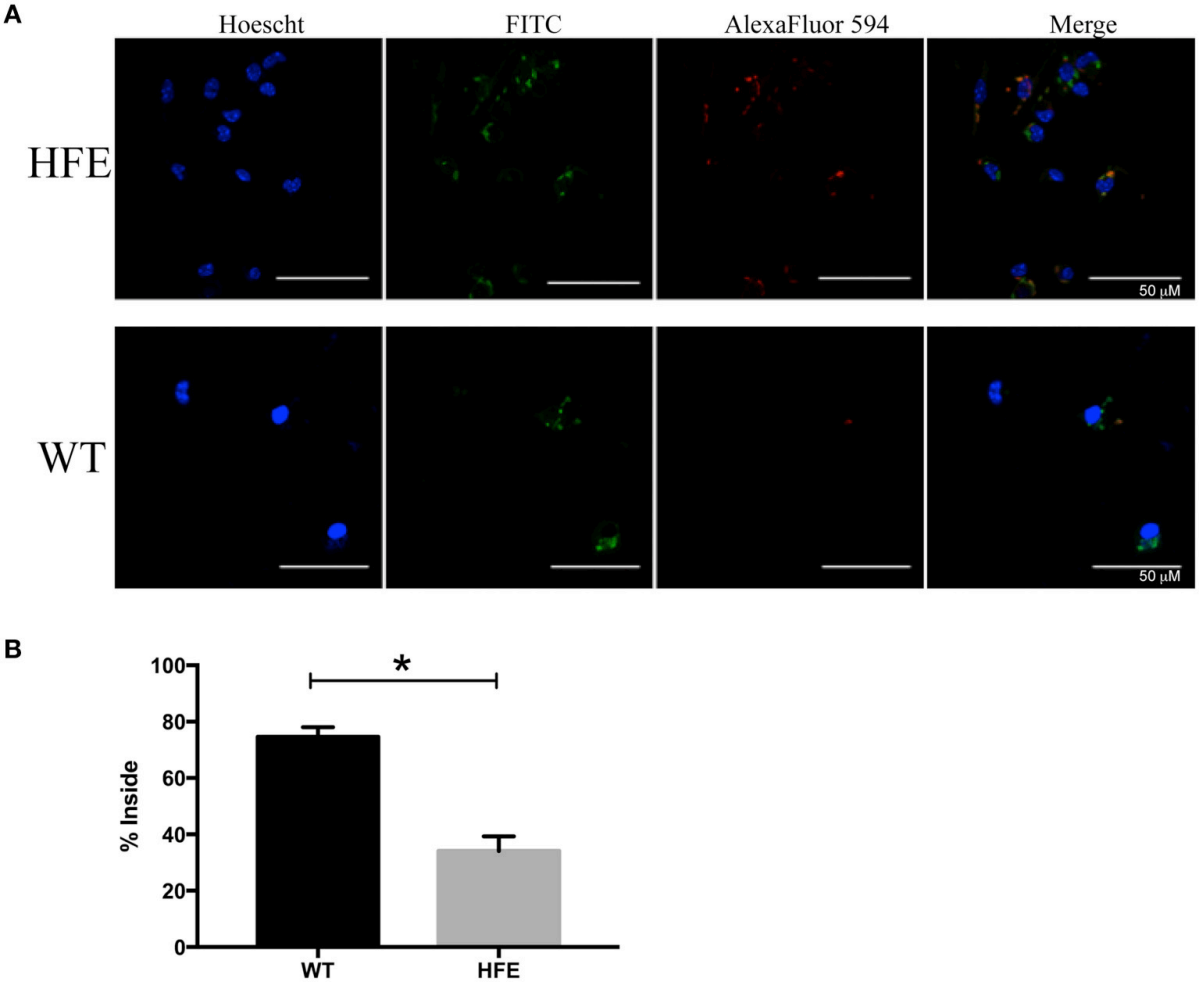
**Peritoneal macrophages isolated from hemochromatosis mice are defective in ***Yersinia*** uptake compared to wildtype macrophages**. Elicited peritoneal macrophages from naïve WT and *Hfe*^*C*282*Y*∕*C*282*Y*^ mice were infected with pYV^−^ *Y. pseudotuberculosis* and percent of bacterial uptake quantitated after 30 min by immunofluorescence microscopy. Extracellular bacteria were stained both red and green, while internalized bacteria were only stained green. **(A)** Representative images are shown in addition to the **(B)** average % internalized bacteria ± standard deviation of 17–20 frames from two biological replicates. ^*^p < 0.02 as determined by a Student *t*-test.

Previous studies indicated that phagocytic cells isolated from human HH patients were defective in phagocytosis (van Asbeck et al., 1984; Moura et al., 1998). As the *Yersinia* T3SS has potent anti-phagocytic activity (Pha and Navarro, 2016), we examined the ability of peritoneal macrophages to take up pYV^−^ *Y. pseudotuberculosis*. Consistent with previous studies, *Hfe*^C282Y∕C282Y^ macrophages contained two-fold fewer intracellular bacteria than wildtype macrophages (Figure 6). These data indicate that the effector function defect of HH mouse phagocytes partly negates the virulence requirement of the *Yersinia* T3SS.

## Discussion

In this study, we demonstrate that two HH mouse models are more susceptible to disseminated infection with fully virulent *Y. pseudotuberculosis* than are wildtype mice, consistent with clinical data on humans with HH. Furthermore, *Y. pseudotuberculosis* lacking the T3SS-encoding virulence plasmid pYV are able to colonize a higher percentage of HH hosts and disseminate into deeper tissues. As peritoneal macrophages isolated from HH mice were less phagocytic toward pYV^−^ *Yersinia* than their wildtype counterparts, we propose that the requirement for the *Yersinia* T3SS, which is strongly anti-phagocytic, is diminished in HH hosts compared to healthy hosts.

Hereditary hemochromatosis is characterized by an increase in intestinal absorption of iron (Hanson et al., 2001). As there is no physiological process to rid the body of this excess iron, it accumulates in organs throughout the body such as the heart, pancreas, and liver, leading to tissue damage and decreased immune response (Hanson et al., 2001; Wang et al., 2008; Hentze et al., 2010; Pietrangelo, 2010; Ekanayake et al., 2015; Rishi et al., 2015). This condition causes an increased susceptibility to serious infection with specific bacterial pathogens including enteropathogenic *Yersinia*. However, it is unclear whether this increase in susceptibility of HH hosts is due to increased virulence of the pathogen, enhanced bacterial fitness due to increased iron availability, or a combination of these factors (Sinkovics et al., 1980; Christopher, 1985; Abbott et al., 1986; Bullen et al., 1991; Mennecier et al., 2001a; Harris and Paraskevakis, 2012). In this study, we demonstrate that two murine models of HH, carrying *Hfe*^C282Y∕C282Y^ or *Hjv*^−∕−^ mutations, display increased susceptibility to *Y. pseudotuberculosis* infection. The more severely iron overloaded *Hjv*^−∕−^ mice were significantly more susceptible to *Yersinia* infection than the *Hfe*^C282Y∕C282Y^ mice with milder iron overload, as *Hjv*^−∕−^ but not *Hfe*^C282Y∕C282Y^ mice or wildtype mice showed overt signs of disease 5 days post-inoculation with 2 × 10^8^ *Y. pseudotuberculosis*, while *Hfe*^C282Y∕C282Y^ mice had higher liver CFU than wildtype mice. This defect in resistance of *Hjv*^−∕−^ mice to *Y. pseudotuberculosis* is consistent with the enhanced susceptibility of these mice to a *pgm*^−^ vaccine strain of *Y. pestis* (Quenee et al., 2012).

Perhaps most intriguing is the finding that the increased bacterial burden observed for HH mice can occur in the absence of the Ysc T3SS, which is required for disseminated *Yersinia* infection of wildtype mice (Cornelis, 2002). Wang et al. described an attenuated inflammatory response of macrophages isolated from HH mice to *Salmonella* (Wang et al., 2008). In addition, macrophages and polymorphonuclear leukocytes from HH humans have been previously shown to have decreased phagocytic and microbicidal properties (van Asbeck et al., 1984; Weiss et al., 1994; Moura et al., 1998; Walker and Walker, 2000). As these host defenses are also targeted by *Yersinia* T3SS effector proteins, it is possible that in HH mice, several host defense pathways that must normally be inactivated by the Ysc T3SS to enable *Yersinia* growth are already compromised as a result of the downstream consequences of iron overload. However, we could not detect any statistically significant differences in production of TNF-α, MCP-1, IFN-γ, or IL-6 in the livers of wildtype and HH mice infected with *Y. pseudotuberculosis*, although the elevated colonization of HH mice compared to wildtype mice complicated this analysis, as cytokine/chemokine level correlated with CFU load. Furthermore, elicited peritoneal macrophages from naïve HH mice actually produced increased levels of TNF-α, MCP-1, or IL-6 in response to *Yersinia* compared to wildtype macrophages. However, we did observe a defect in the ability of HH peritoneal macrophages to take up *Yersinia* compared to wildtype macrophages. Therefore, it is possible that the phagocytic properties of tissue macrophages in the HH mice are compromised, rendering the anti-phagocytic activity of the Ysc T3SS less important in these tissues. Interestingly, there was no decrease in Ly6G^+^ cell infiltration in infected *Hjv*^−∕−^ mouse livers compared to infected WT livers (data not shown). Collectively, these data suggest that phagocytes are recruited to sites of infection in HH animals and produce cytokines in response to microbial PAMPS, yet are ineffective in microbial uptake.

Our data support the proposed model that an increase in iron availability might dampen T3SS expression through IscR control of the T3SS master regulator LcrF (Miller et al., 2014), as loss of the T3SS in iron overloaded mice would not be as detrimental to *Y. pseudotuberculosis* virulence as it is in wildtype mice. Furthermore, with increased emphasis on identification of novel antimicrobial strategies and research on chemical inhibitors of the T3SS as virulence blockers, our data suggests that, while T3SS inhibitors may one day be used to prevent or treat *Yersinia* infection, they may not be effective in the context of host iron overload. Recently there was a case of lethal laboratory-acquired plague in a researcher with undiagnosed HH who became infected while working with pgm^−^ *Y. pestis*, a strain with diminished virulence (Quenee et al., 2012). *Yersinia* strains lacking the T3SS are also considered to have decreased pathogenic potential; however, based on the data presented here, HH hosts may be more susceptible to these strains as well.

Huang et al. showed that *Hjv*^−∕−^ mice contained less splenic non-heme iron compared to wildtype mice, in contrast to the elevated non-heme iron observed in *Hjv*^−∕−^ livers (Huang et al., 2005). Quenee et al. also found iron deposits in the livers, but not the spleens, of *Hjv*^−∕−^ mice. Macrophages play an important role in the recycling of iron from senescent red blood cells, and Huang et al. demonstrated that the increased iron recycling in *Hjv*^−∕−^ mice leads to higher extracellular iron and lower macrophage intracellular iron (Huang et al., 2005). As the spleen contains numerous macrophages (Cesta, 2006), it is possible that decreased macrophage iron in *Hjv*^−∕−^ mice contributes to the lower overall splenic iron level (Hentze et al., 2010). Interestingly, we observed an increase in bacterial burden in both the liver and spleen of *Hjv*^−∕−^ mice compared to wildtype mice. As we did not perform perfusion prior to organ harvesting, as per standard practice in the field, it is possible that enhanced bacterial colonization of *Hjv*^−∕−^ spleens reflects enhanced *Y. pseudotuberculosis* growth in *Hjv*^−∕−^ blood. Unlike *Hfe*^C282Y∕C282Y^ mice, which have been shown to have comparable serum iron concentrations relative to wild type mice, *Hjv*^−∕−^ mice have elevated iron levels in the blood relative to wild type (Zhou et al., 1998; Gkouvatsos et al., 2014). Indeed, *Hjv*^−∕−^ mice displayed enhanced *Yersinia* spleen colonization but *Hfe*^C282Y∕C282Y^ mice did not.

Interestingly, Quenee et al. showed that a recombinant protein vaccine that targets the T3SS needle tip protein LcrV protects *Hjv*^−∕−^ mice against fully virulent plague or against a live, attenuated vaccine strain of *Y. pestis* (Quenee et al., 2012). The rV10 vaccine was previously shown to elicit antibodies to LcrV, and anti-LcrV antibodies are known to inhibit translocation of Yop effectors into host cells (DeBord et al., 2006; Quenee et al., 2010). These data suggest that anti-LcrV antibody targeting of *Y. pestis* protects HH mice against otherwise lethal plague either by inactivation of the T3SS and/or through opsonization of *Yersinia* (Quenee et al., 2010). Given our data showing that the *Y. pseudotuberculosis* T3SS is dispensable for disseminated infection, it is possible that the increased bacterial load that we observed also occurs in the model of survival utilized by Quenee et al. but that the HH mice are able to clear the infection.

Our findings as well as those of Quenee et al. (2012) suggest that the increased susceptibility of HH hosts to *Yersinia* infection is likely a result of excess iron available to promote bacterial growth. Indeed, *Y. pestis* strains lacking the ability to synthesize yersiniabactin, an iron scavenging siderophore, are fully virulent in HH hosts (Quenee et al., 2012). Additionally, mice infected with *Y. enterocolitica* or *Y. pestis* that were given either iron-dextran or the siderophore Desferal, which can be used by *Yersinia* as an iron source, displayed reduced lethal doses as well as a more severe yersiniosis (Burrows and Jackson, 1956; Robins-Browne and Prpic, 1985; Galvan et al., 2010; Quenee et al., 2012). Collectively, these results and the data shown here suggest that the progression and pathology of yersiniosis may differ greatly in iron overloaded vs. non-iron overloaded hosts because of excess bioavailable iron, deficiency in the phagocytic properties of immune cells, and differences in bacterial virulence factors required to cause disease.

## Author contributions

HM and LS contributed equally to this work. VA and HM designed the study. HM, LS, and WA performed the experiments. HM and VA wrote the paper.

### Conflict of interest statement

The authors declare that the research was conducted in the absence of any commercial or financial relationships that could be construed as a potential conflict of interest.
